# Linkers of Cell Polarity and Cell Cycle Regulation in the Fission Yeast Protein Interaction Network

**DOI:** 10.1371/journal.pcbi.1002732

**Published:** 2012-10-18

**Authors:** Federico Vaggi, James Dodgson, Archana Bajpai, Anatole Chessel, Ferenc Jordán, Masamitsu Sato, Rafael Edgardo Carazo-Salas, Attila Csikász-Nagy

**Affiliations:** 1 The Microsoft Research-University of Trento Centre for Computational Systems Biology, Rovereto, Italy; 2 The Gurdon Institute, University of Cambridge, Cambridge, United Kingdom; 3 Department of Biophysics and Biochemistry, University of Tokyo, Tokyo, Japan; University of Heidelberg, Germany

## Abstract

The study of gene and protein interaction networks has improved our understanding of the multiple, systemic levels of regulation found in eukaryotic and prokaryotic organisms. Here we carry out a large-scale analysis of the protein-protein interaction (PPI) network of fission yeast (*Schizosaccharomyces pombe*) and establish a method to identify ‘linker’ proteins that bridge diverse cellular processes - integrating Gene Ontology and PPI data with network theory measures. We test the method on a highly characterized subset of the genome consisting of proteins controlling the cell cycle, cell polarity and cytokinesis and identify proteins likely to play a key role in controlling the temporal changes in the localization of the polarity machinery. Experimental inspection of one such factor, the polarity-regulating RNB protein Sts5, confirms the prediction that it has a cell cycle dependent regulation. Detailed bibliographic inspection of other predicted ‘linkers’ also confirms the predictive power of the method. As the method is robust to network perturbations and can successfully predict linker proteins, it provides a powerful tool to study the interplay between different cellular processes.

## Author Summary

Analysis of protein interaction networks has been of use as a means to grapple with the complexity of the interactome of biological organisms. So far, network based approaches have only been used in a limited number of organisms due to the lack of high-throughput experiments. In this study, we investigate by graph theoretical network analysis approaches the protein-protein interaction network of fission yeast, and present a new network measure, linkerity, that predicts the ability of certain proteins to function as bridges between diverse cellular processes. We apply this linkerity measure to a highly conserved and coupled subset of the fission yeast network, consisting of the proteins that regulate cell cycle, polarized cell growth, and cell division. In depth literature analysis confirms that several proteins identified as linkers of cell polarity regulation are indeed also associated with cell cycle and/or cell division control. Similarly, experimental testing confirms that a mostly uncharacterized polarity regulator identified by the method as an important linker is regulated by the cell cycle, as predicted.

## Introduction

The eukaryotic cell cycle is one of the most important and evolutionary conserved processes of cells [1], [2]. The cell cycle integrates signals from multiple pathways to control tissue growth and homeostasis in multicellular organisms, as well as reproduction and proliferation in single cell organisms [3]. To ensure cell integrity, the cell cycle regulates and is regulated by other key processes such as DNA replication, cytokinesis and cell growth [4]–[9]. Disruption of the regulation between the cell cycle and other cellular processes can cause a myriad of cellular pathologies including defects in cell shape, abnormal cell growth and aneuploidy, potentially leading to cancer [10].

With the accumulation of data from high-throughput biology as well as the generalisation of manually curated online databases, we now can mine existing biological networks to make experimentally verifiable predictions about system-wide properties of genes and gene products. In this work, we present a new method to search for proteins that serve as linkers between distinct functional sub-networks. Because of the well-characterized interactions between the cell cycle and other processes in the fission yeast *Schizosaccharomyces pombe*, we focus our analysis on this organism, where these processes have not yet been investigated yet by protein interaction network analysis methods.

The fission yeast - a rod-shaped unicellular eukaryote - is ideally suited to study the relationship between cell cycle and cell polarity regulation, as its highly polarized growth pattern is tightly correlated with cell cycle progression [7], [11]. After cytokinesis, newborn *S. pombe* cells resume growth in G1 in a monopolar fashion from their ‘old end’ - the cell end that existed prior to division - and later in early G2 activate growth at their ‘new end’ derived from the site of septation, an event termed new-end take-off or NETO [12]. Bipolar growth then continues through G2 until cells reach a critical size, after which cells enter M phase again. At that point cells stop growing [13], mitosis takes place and each cell divides by growing a septum in its middle. Daughter cells resume their cyclic pattern of growth at the ends and division at the middle, a pattern that relies on the cytoskeleton of actin and microtubules and on diverse polarity-regulating proteins (‘polarity factors’). Cytokinesis, polarity, and the cell cycle have been extensively studied in fission yeast –using both experiments and mathematical modelling [14]–[18]. The insights gained from studies in fission yeast often carry over to higher eukaryotes, as the molecular machinery controlling those processes has been highly conserved throughout evolution [1], [19], [20].

Several proteins have been identified that play important roles connecting these processes in fission yeast. For example, the polarized growth-regulating DYRK kinase Pom1 [21] was recently shown to form a spatial gradient that is used by the cell cycle machinery to sense the length of the cell [17], [22], [23]. Another link was observed between the morphogenesis-related NDR kinase network (MOR) and the septation initiation network (SIN) [24]. MOR is important for the localization of actin patches to sites of polarized growth, while SIN is responsible for triggering cytokinesis. It was discovered that SIN inhibits the MOR pathway, through inhibition of the Orb6 activator Nak1. MOR itself also interferes with SIN, and this antagonism is required for proper progression through the cell cycle [25], [26]. Furthermore, a similar antagonism between the MOR and SIN pathways has also been observed in higher eukaryotes [27], [28]. The NETO transition from monopolar to bipolar growth and the switch from polarized growth to actin ring-mediated cell septation are also controlled by the cell cycle [13], thus the cell cycle machinery enforces a major control on both polarized growth and cytokinesis. Although many polarity or cytokinesis regulators contain potential phosphorylation sites for the cell cycle-regulating Cyclin-Dependent Kinases [29] (CDK), the molecular details of these couplings are not well known. In the other direction, if either polarized cell growth or cytokinesis is inhibited, both can send signals to stop the cell cycle [30], [31], further underlining that these three functional modules are highly interlinked.

To tackle the interplay between different cellular processes, we utilized a network theory approach. Hitherto, network based approaches have only been used in a limited number of organisms, due to the paucity of genome-wide interaction data available for most species. Recently, however, improvements in automatic experimental annotation, literature mining [32], machine learning [33] and orthology annotations [34], are allowing the use of network approaches in a wider range of organisms. For example, ‘meta databases’ such as STRING [35], [36], benchmark information from multiple sources and provide for each possible interaction a confidence score that reflects the likelihood of a set of proteins of actually interacting. Here, we take advantage of such developments and build on the efforts of the fission yeast community in annotating protein functions [37]–[39], to establish a new method to identify proteins linking diverse cellular processes, based on integrating Gene Ontology (GO) [40], [41] and Protein-Protein Interaction (PPI) data together with network theory based measures. Network-based approaches in biology have been used in the past to identify community structures, study lethality, identify specific regulatory circuits and study hierarchical organization [42]. In particular, the nature of large scale protein-protein interaction networks has recently been under considerable debate with different groups disagreeing about the modularity of networks, as well as the properties of the nodes responsible for bringing together different modules [43]–[46]. In this work, we sidestep the difficult problem of identifying hierarchical modules in a large, genome-wide network and focus instead on a method to identify proteins that link different cellular processes. To do this, we use the highly characterized sub-genomic network consisting of proteins regulating the cell cycle, cytokinesis, and polarized cell growth in fission yeast. We propose a new network measure, termed ‘linkerity’, and use it to predict a novel role for a number of proteins as key bridges between these biological processes.

## Results

### Constructing and validating the fission yeast protein interaction network

We constructed the fission yeast protein-interaction network using data from STRING [35], [36] and BioGRID [47]. By applying a cutoff on the confidence score from STRING, we can reject interaction pairs for which there is a limited amount of evidence (see Materials and Methods for details on data in STRING) and use the remaining edges to construct a non-directed and non-weighted network.

**Figure 1:**
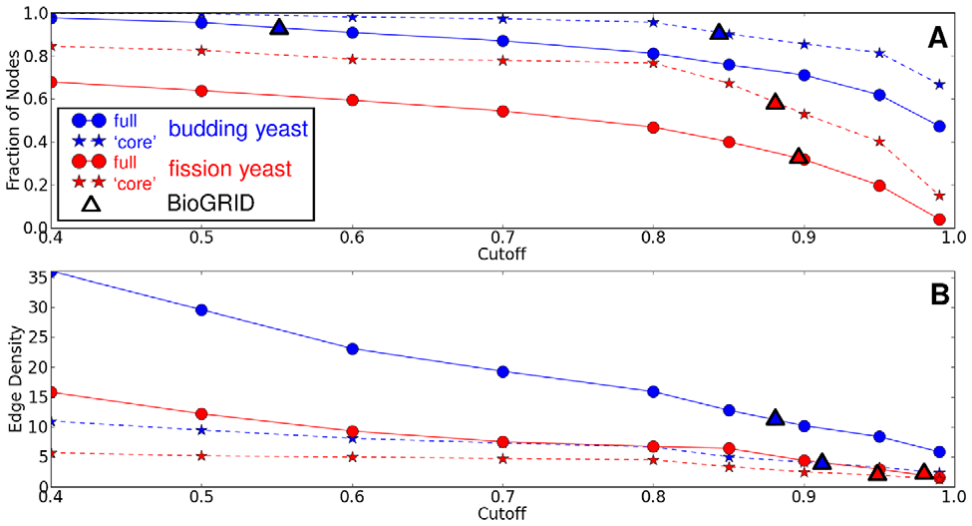
Dependence of network measures on protein-protein interaction data quality. As we increase the minimal accepted confidence (cutoff) for the PPI data of the STRING database, the number of nodes in the largest connected component (**A**) and the network density (**B**) both decrease for all networks. This decrease is faster in fission yeast compared to budding yeast, and faster in the full organism network compared to the core network. Triangles overlaid on each curve show the same network measures for the PPI network based on the BioGRID database, the position on the x-axis of BioGRID data is calculated using linear interpolation to estimate the corresponding cutoff in STRING which would give a similarly-sized network, thus the overlay of the BioGRID data gives an indication how this relates to different cutoff STRING data. As can be seen from the figure panels the fission yeast core network is quite robust to cutoff changes and behaves similarly to the core network of budding yeast cells. This is also true for the core networks based on BioGRID data.

**Figure 2:**
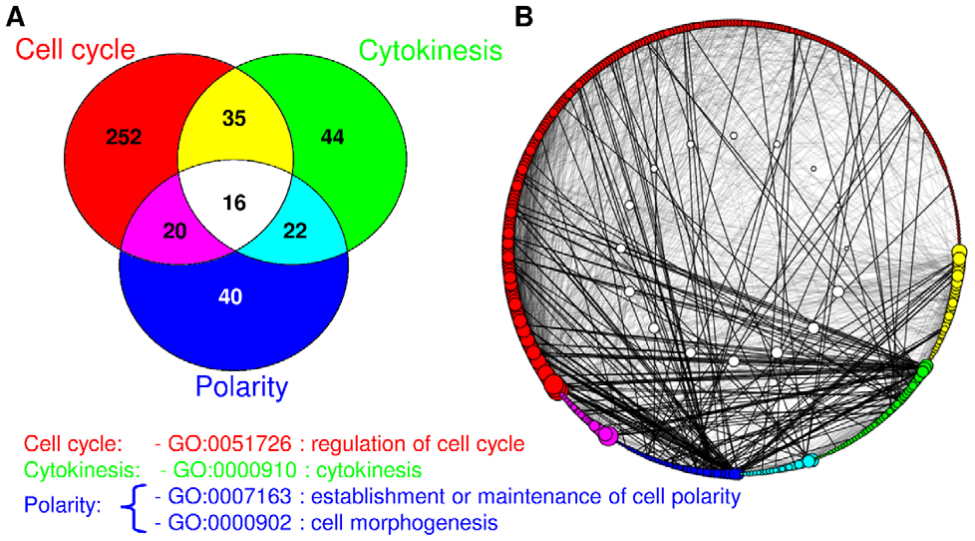
The cell cycle + cytokinesis + polarity = core interaction network of fission yeast proteins. (**A**) Venn diagram showing the overlap among the different Gene Ontology functional groups for the proteins belonging to the core network. Proteins with multiple functional annotations have colours that are the sum of the colours of the individual functional annotations; proteins belonging to all three functional groups are in white. (**B**) Protein-protein interactions inside the fission yeast core network (from the STRING database at cutoff 0.7). Node colours are the same as in panel A. Node size is proportional to the degree of each protein, and node order within a category (clockwise) is also determined by degree. 165 black edges link proteins that do not share functional annotations, while 1869 grey edges link proteins that have at least one common GO annotation (thus white nodes have only grey links). White nodes (nodes belonging to all categories) are shown in the inner circle in the middle of the network.

We then examined the effects of increasing the cutoff in STRING confidence scores in both the genome-wide interaction dataset of fission yeast and that of the better characterized budding yeast *Saccharomyces cerevisiae* on the network topology. Increasing the cutoff decreased the amount of nodes (Figure 1A) and the edge density (Figure 1B) in the largest component (the connected component in the network containing the highest number of edges and nodes) of both the fission and budding yeast networks (Tables S1, S2). This decrease was less sharp in budding yeast compared to fission yeast due to the extensive amount of genome-wide interaction experiments carried out in the former, increasing the amount of high-confidence interactions. Interestingly, in the ‘core’ sub-network consisting of proteins involved in cell cycle regulation, polarity and cytokinesis (Figure 2 for fission yeast and Figure S1 for budding yeast), the drop off in the number of nodes and edges was far less significant in both yeasts, suggesting that interaction data for the core fission yeast network tends to be more reliable than interaction data for the rest of the network (Figure 1, red stars versus red dots, also Tables S1, S2, S3, S4). As a more stringent test, we constructed networks for both organisms using only data from BioGRID [47]. BioGRID is a database that only contains data from manually annotated experiments (distinguishing between experiments that show direct physical interaction and genetic interactions). Networks built using the BioGRID physical interaction data also show that the core networks of fission yeast and budding yeast are relatively dense, while the fission yeast organism-wide network is rather sparse (Figure 1). Even with the relatively high coverage of the core (regulation of cell cycle, cytokinesis, polarity) network in fission yeast, it is important to note that fission yeast lacks any genome-wide protein-protein interaction experiments, and as such, several of the interactions predicted by STRING are based on indirect evidence such as genetic interactions, inference from homology, or literature mining [35], [36].

As no analysis of the fission yeast network has been previously published, we performed a few checks to verify that our network construction procedure was giving sensible results, and that the data for fission yeast available in STRING was of sufficiently high quality. As a first check, we sought to replicate a number of analyses previously performed with budding yeast (Table 1). At a cutoff of 0.7 (defined by STRING as a ‘high confidence’ threshold), the genome-wide fission yeast network has 2770 nodes with at least one connection and 20432 edges compared to 5477 nodes and 105429 edges found in budding yeast, although they have approximately similar number of proteins. We calculated the degree distribution for the nodes in the network, and observed that, as previously described for numerous other complex networks [48], the fission yeast PPI network has a scale-free distribution (Figure S2). We also repeated analyses done in numerous other studies examining the relationship between network measures and gene deletion lethality [43]–[45]. As reported for budding yeast, we observed that degree (the number of interactions with other proteins) is the best predictor network measure of gene deletion-induced lethality in fission yeast, and that ratio of these essential genes among hubs (the top 20% of proteins by degree) is even higher in fission yeast than in budding yeast (see Text S1).

**Table 1:** Network statistics and gene essentiality comparison between the two yeasts.

	budding yeast	fission yeast	references
**Degree Distribution:**	Scale Free	Scale Free	[105]
**BC Distribution:**	Scale Free	Scale Free	[106]
**Network measure most predictive of lethality:**	Degree	Degree	[45], [107]
**% of essential genes in hubs**	39	56	[45]
**% of essential genes in bottlenecks**	31	47	[45]

Quality check of the fission yeast PPI network in comparison to earlier published data on the budding yeast PPI network. Hubs are the top 20% of nodes in the network according to degree. Bottlenecks are the top 20% of nodes in the network according to betweenness centrality (BC).

Since there is no high-throughput genome-wide interaction data available for fission yeast, we tested the possibility that highly investigated proteins might have more interactions. To check this, we tested to see whether the number of abstracts in PubMed discussing a particular protein was correlated with the degree of that protein in the network. The Pearson correlation between the number of PubMed abstracts citing a protein and its degree in the network was 0.13 for budding yeast (p-value<10^−19^) and 0.14 for fission yeast (p-value<10^−13^) (details in Tables S1, S2), suggesting there is no fission yeast-specific bias for proteins with large amounts of publications in STRING networks. However, a large amount of evidence for the fission yeast interactions in STRING is obtained from homology, and specifically from interactions of homologues proteins in budding yeast. As essential genes are more likely to be conserved [49], [50] and STRING is more likely to identify homology between highly conserved genes, it is possible that this might introduce a subtle bias making essential genes appear to be more highly connected in virtue of their higher conservation. This is consistent with the observation that a very high percentage of hubs in fission yeast appear to be essential (Text S1).

### The core network of regulators of the cell cycle, cell polarity and cytokinesis

The sub-network of all proteins regulating cell cycle, cytokinesis and polarized growth, henceforth, the ‘core’ network (see Materials and Methods for definitions of exact GO terms used) in fission yeast contains 550 proteins: 384 of those are associated with regulation of cell cycle, 155 with cytokinesis and 139 with polarity. Using a cutoff of 0.7 in STRING, 429 of the total 550 proteins are connected to the largest connected component of the core network. Most of the proteins not in the network have no known interactions, and the second largest connected component contains only 4 proteins, thus we focus only on the interaction network of the largest connected component. There are a high number of proteins with multiple functions in the network (Figure 2A), 16 of them (Alp4, Cdc15, Gsk3, Lsk1, Mor2, Orb6, Pab1, Pmo25, Pom1, Ppb1, Ras1, Scd1, Shk1, Sid2, Tea1, Wsp1) are important for all three cellular processes and 77 have dual functions. The ratio of multifunctional proteins is quite similar to the ratio in the analogous core budding yeast network (Figure S1). Interestingly the budding yeast core network contains less nodes than the fission yeast core network (although it is more densely connected), this could be a consequence of the extensive studies of cytokinesis [19], cell cycle [51] and cell polarity [13] and their careful annotation in fission yeast [37]–[39], but it also reflects the loss of some of the conserved eukaryotic cell cycle genes from budding yeast [29], [52].

The core interaction network contains several interactions between proteins that do not share a GO annotation; however the majority of links (91%) are between proteins which share at least one functional annotation among those under consideration (regulation of cell cycle, cytokinesis, and polarity) (Figure 2B). To probe this, we examined the relationship between the functional annotation of a node and that of its interaction partners. In fission yeast, any protein with a given functional annotation was 11 times (1.9 would be expected randomly, see Figure S3A) more likely to interact with another protein with the same functional annotation than with another protein with different functional annotations (for the budding yeast core network, this ratio was 4.5 vs. 1.06 expected, see Figure S3B). Since fission yeast has more proteins that belong to all three categories (16 in fission yeast versus 6 in budding yeast), we tested to see whether this observed functional modularity was due to their presence. We removed all proteins belonging to all three categories from both networks and repeated the analysis. This did not significantly alter the results as the ratios remained after the removal (10.38 times more likely for fission yeast and 4.16 for budding yeast) suggesting that the functional modularity observed in fission yeast is not caused by the presence of highly connected proteins with multiple annotations, but rather that the fission yeast network is characterized by strong connections between local communities that share functional annotations. It is however important to note that the GO categories ‘regulation of cell cycle’ and ‘cytokinesis’ are partially overlapping. In particular ‘regulation of cell cycle cytokinesis’ is a child term of both ‘regulation of cell cycle’ and ‘cytokinesis’. Even when taking this overlap into account in the analysis, we still observe a high degree of functional modularity in the core networks of both fission and budding yeast (not shown).

**Figure 3:**
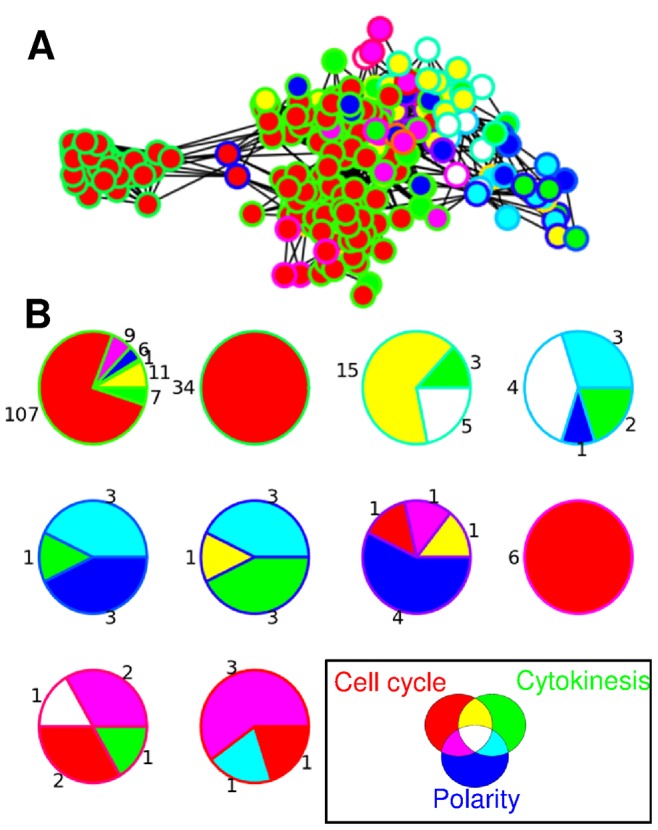
Segregation of functional communities in the core network. A clique propagation algorithm was used to identify locally highly connected communities of the core network. The ten cliques generated by the algorithm segregate in the interaction network if laid out by a force-based algorithm that brings closer together the stronger interacting groups (**A**). Node colour determined by the functional annotation (same as Figure 2, inset on panel B here). Proteins belonging to the same clique share the same border colour. Proteins belonging to the same clique largely share functional annotations. Pie charts show the functional distribution of proteins found in each clique (**B**). Numbers report the number of proteins with the annotations corresponding to the given colour coded annotation (see inset for colours).

We further analyzed this effect using a community detection algorithm, which identifies local communities in a network and allows their overlap – as we have nodes with multiple annotations. We applied the k-clique propagation algorithm [53], [54] and examined the communities generated by the method with k = 4. While the communities generated by the algorithm do not exactly match the functional annotations, we find that the cliques generated by the algorithm are primarily formed by proteins that share functional annotations (Figure 3A,B). Upon closer examination, the few proteins that do not share a functional annotation with the other members of a clique seem to have related roles: for example, in the 5^th^ clique on Figure 3B, the lone ‘non-polarity’ protein is Rgf3, which was shown to play an important cell-wall remodeling role downstream of Rho1, one of the key regulators of polarity [55], [56] (consult Table S5 for all clique members).

### Identification of ‘linker’ proteins by network analysis

**Figure 4:**
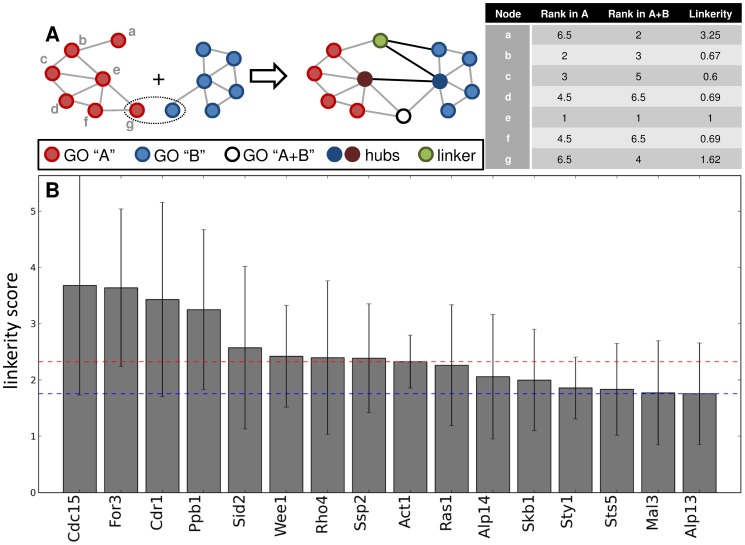
Concepts of ‘linker’ protein detection and robustness of the method. (**A**) ‘Linker’ proteins are found at the edge of a sub-network, but are central in the context of a larger network. Such proteins have low betweenness centrality (BC) score when considered in the context of their sub-network, but have a high BC score in the core network even though they do not have a functional annotation to the other category making up the core network. Black edges indicate edges between proteins that do not share functional annotations, while the other edges are gray. Table on right gives ranks and linkerity measures for all nodes in network ‘A’ in the same style as Table 2 does. (**B**) Analysis of the robustness of linkerity scores for the polarity network of fission yeast cells. We added 10% extra edges randomly to the network, and computed the linkerity score of all proteins after each iteration. Bars show mean ranking with standard deviation. Blue dashed line indicates cutoff for top 10% and red line marks the top 20% (results of other type of network perturbations are reported in Figure S5).

To systematically study proteins linking different cellular processes, we next used a network-based approach aiming to identify proteins that function as ‘linkers’ between different functional categories (Figure 4A). To do so, we constructed protein-protein interaction networks consisting only of proteins with one of the investigated functional annotations (cell cycle, cytokinesis or polarity regulation). We then calculated the betweenness centrality score for every node in each of these networks and in the merged core network. Betweenness Centrality (BC) measures how often a node is found in the shortest path between pairs of other nodes in the network; intuitively, it can be thought of as a measure of how central a node is in a network. If a node has a low centrality score it is localized at the fringe of a network, while if it has a high score it is localized near the centre. Next we ranked the proteins based on their BC score (in case of a tie, these proteins got their average rank). To ensure that this ranking method is robust even in the presence of imperfect interaction data certainly missing important links, we randomly added 10% extra edges to all the networks 1000 times, and recalculated the ranking of all proteins at each iteration (Figures S4). While the exact ranking of proteins is not very robust to addition of extra edges, if we examine all the proteins in the top 20%, we can observe that most fluctuate out of the top 20% only very rarely, and that we nearly never observe a protein in the top 10% drop out of the top 20%. It is also reassuring that the top of the rankings starts with expected key regulators of each function: the polarity landmark Tea1 [57]–[59], the actin-regulating Rho GTPase Cdc42 [60], [61] and actin (Act1) all came on the top of the polarity list. At the same time Cdc2, Wee1 and Cdc25 [62] are on the top of the cell cycle list (and also on the top of the core list) and the SIN scaffold Cdc11 [63] and the CDK counteracting, SIN activator phosphatase Clp1 [64], [65] are leading the cytokinesis ranking (Figure S4 and Table S3).

In the next step we compared the betweenness centrality rank of every protein in a sub-network to its relative rank in the core network. Only proteins that were originally in the sub-network were considered during this ranking based on scores they got for their position in the core network. We then calculated the ratio of the relative rank in the core network and the rank in the sub-network. We termed this calculated value ‘linkerity’, as this value is high for proteins that are found at the fringe of the network of proteins controlling a given cellular process, but central when considered in the context of a bigger network (Figure 4A):
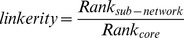
(1)Proteins with high linkerity, we hypothesized, are likely to play a crucial role to function as linkers between different cellular processes. Specifically, we focused on the relationship of the polarity network to the rest of the core network to clarify how the cell cycle and the cytokinesis machinery control the temporal changes in the localization of polarized growth zones (top of Table 2, consult Table S3 for the rest of the list). Here, we show the top 10 proteins with the highest linkerity scores. These proteins became far more central when the polarity sub-network was embedded into the core network. Most of these proteins have GO annotations for multiple processes (among the annotations under consideration), thus their linking capacity is not that surprising. Novel linkers of polarity regulation could be those that were not associated with cytokinesis or cell cycle control but gained a high linkerity score in our analysis. The formin For3 [66], the AMP-activated, Snf1-like protein kinase Ssp2 [67], [68], the RNB-like protein Sts5 [69] and the MRG family protein Alp13 [70] are examples of proteins that match this. For3 is a well-characterized regulator of Tea1 to Cdc42 signalling [71], [72], the other three are less well characterized. The Rho GTPase Rho4 [73] might be also an interesting linker candidate as it has established roles in polarity and cytokinesis regulation, but its exact function is not well characterized and it has no association to cell cycle regulation. Despite this, Rho4 has a central position in the core network that contains 75% cell cycle proteins (Figure 2A), furthermore its expression is cell cycle regulated [74]. The highest linkerity proteins from the cytokinesis and cell cycle regulation networks also contain a number of proteins which are also associated with polarity regulation (Table 2). Scd1, Pom1 and Tea1 are on the top of the cell cycle linkerity list and Pmk1 [75], Shk1 and Tea1 lead the cytokinesis list after Bgs1, which is essential for cell wall synthesis [76], but has no polarity related GO annotation. These are on the edge of the cell cycle regulation or cytokinesis network but became central when they are merged with the polarity network, thus these can be also considered as linkers. As above for BC scores, we analysed the robustness of linkerity in the presence of imperfect network interaction data: we added or removed 10% of the edges from the core network at random or following a preferential attachment model and calculated linkerity scores for all proteins. Figure 4B reports the average and standard deviation from 500 random networks with 10% extra edge (other cases in Figure S5) for the top linkerity polarity proteins. Importantly the top 10 of the unperturbed list (Table 2) can be found in the top 16 of the list after 10% possible missing links were considered (Figure 4B).

**Table 2:** Top ten proteins with highest Linkerity measures from the three sub-networks.

Protein Name	GO terms	Rank_Sub_	Rank_Core_	Linkerity
**Polarity proteins**				
Rho4	Pol, Cyt	73.5	8	9.19
For3	Pol	35	5	7
Ssp2	Pol	73.5	19	3.87
Skb1	Pol, CC	38	13	2.92
Sts5	Pol	25	9	2.78
Cdr1	Pol, CC	73.5	29	2.53
Act1	Pol, Cyt	5	2	2.5
Cdc15	Pol, Cyt, CC	57	23	2.48
Alp13	Pol	54	22	2.45
Ppb1	Pol, Cyt, CC	27	12	2.25
**Cytokinesis proteins**				
Bgs1	Cyt, CC	12	3	4
Pmk1	Pol, Cyt	64	16	4
Shk1	Pol, Cyt, CC	15	4	3.75
Tea1	Pol, Cyt, CC	32	9	3.55
Rho4	Pol, Cyt	17	7	2.43
Pab1	Pol, Cyt, CC	67	29	2.31
Cdc7	Cyt, CC	29	13	2.23
Plo1	Cyt, CC	24	11	2.18
Klp5	Cyt	30	14	2.14
Fin1	Cyt, CC	97	46	2.11
**Cell cycle proteins**				
Scd1	Pol, Cyt, CC	288	39	7.38
Pom1	Pol, Cyt, CC	184	26	7.08
Tea1	Pol, Cyt, CC	143	25	5.72
Bgs1	Cyt, CC	30	8	3.75
Cdc10	CC	67	18	3.72
Cdc15	Pol, Cyt, CC	179	63	2.84
Cdc13	CC	15	6	2.5
Its3	Cyt, CC	234	94	2.49
Mal3	Pol, CC	123	50	2.46
Pmh1	Pol, Cyt, CC	76	34	2.23

Proteins were ranked according to BC in the polarity/cytokinesis/cell cycle regulation sub-networks (Rank_Sub_ column) as well as in the core network (Rank_Core_ column). Proteins with the same BC score were given the same ranking. In the core network, we considered proteins that also belonged to the investigated sub-network and skipped all other proteins (thus we had three different core network rankings). The cell cycle network gives higher linkerity scores, since it contains more nodes, thus higher ranking jumps are possible. Consult Table S3 for the rest of the lists. Table S4 contains the same data for budding yeast cells. The second column gives the GO annotations of each protein among polarity (Pol), cytokinesis (Cyt) and cell cycle (CC) related GO terms as defined on Figure 2.

As discussed above, in both fission yeast and budding yeast, we observe a high degree of functional modularity, i.e. proteins tend to interact with proteins that share their functional role. Since linker proteins play a special role in bringing together different cellular processes, we examined whether proteins with high linkerity interacted with proteins with different functional roles at a higher rate than low linkerity proteins. For all the proteins of the core network we calculated the number of its interactors (network neighbours) with cell cycle, cytokinesis and polarity annotations (Table S3). Then for every protein in each functional category (Figure 2) we calculated the ratio of the number of its interactions with proteins with the two other functional annotations to the number of its interactions with proteins with the same functional annotation. We observed that high linkerity is significantly correlated with having a high ratio of heterogeneously annotated neighbours across all functional categories in both yeasts, suggesting that linker proteins do play an important role in bridging proteins from different functional groups (see Text S2 for details).

### Sts5 is a novel linker protein bridging cell polarity to cell cycle

**Figure 5:**
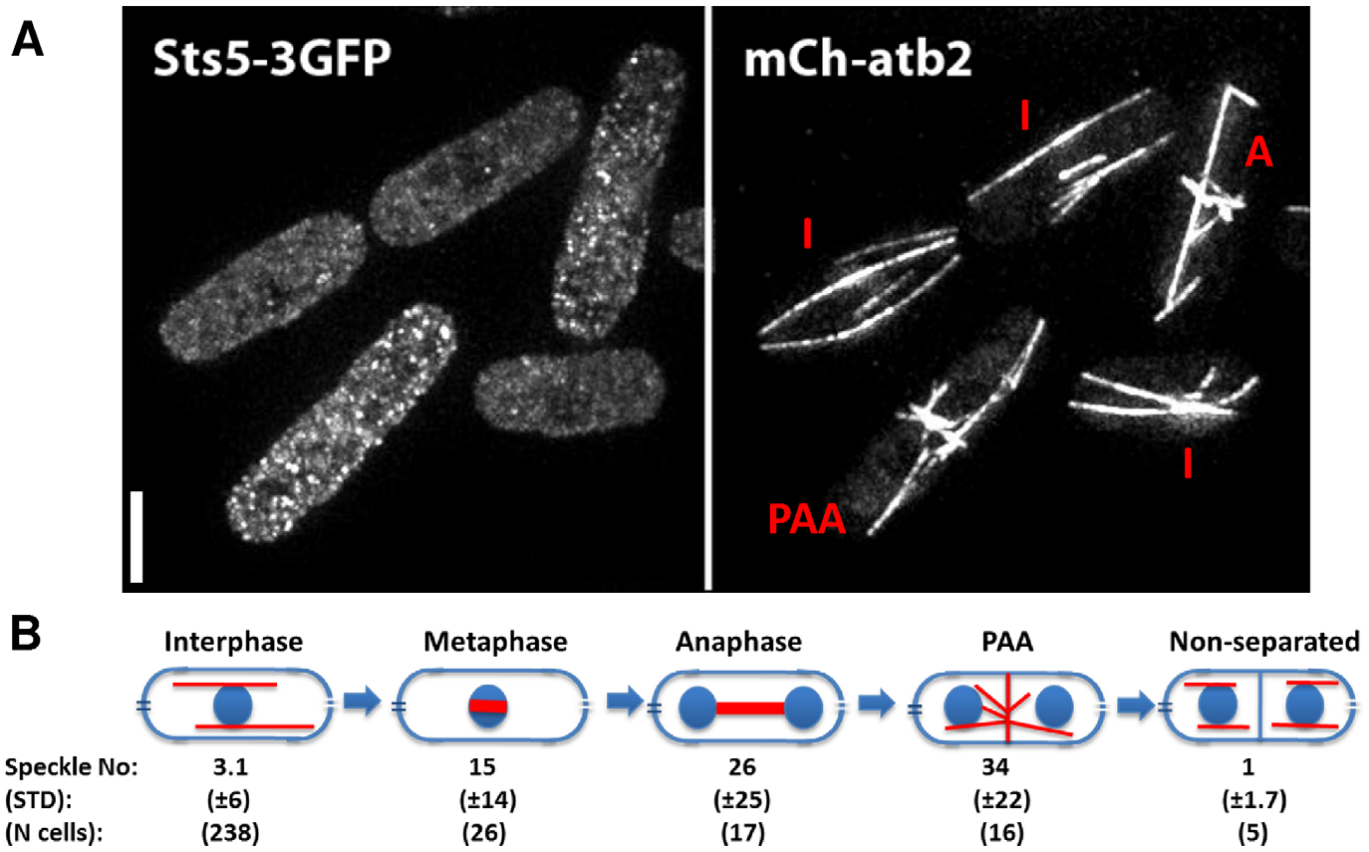
Localization in cells of Sts5 during the cell cycle. (**A**) Imaging of fission yeast cells co-expressing Sts5-3GFP and mCh-atb2 (labelling the different microtubule structures seen through the cell cycle, and hence acting as cell cycle stage indicators). Interphase cells (I) have diffuse Sts5 localization (with a few cytoplasmic speckles) while cells in mitosis (either in anaphase (A) or during the time of the post anaphase array (PAA)) have several Sts5 cytoplasmic dots. Scalebar: 5 µm. (**B**) Population based analysis of cycling cells revealed that at metaphase the number of Sts5 speckles greatly increases and sharply drops during septum formation. Average and standard deviation of number of dots were automatically detected in multiple cells (see Materials and Methods for details).

Among predicted linker proteins we focused on Sts5, which is known to genetically interact with Ssp2 [69], which itself is likely to be linked with the cell cycle machinery as *ssp2Δ* cells cannot start mitosis when nutrient-starved [77]. Sts5 is an orthologue of budding yeast SSD1 [78] and therefore a candidate translational repressor. It is reported to control actin localisation in interphase and *sts5Δ* was shown to be compensated by mutations in Ssp2. Furthermore, Sts5 mRNA levels were shown to oscillate [74], [79]. To examine the interplay between Sts5 and the cell cycle, we tagged the endogenous protein with a triple GFP tag and visualized its localization together with that of mCh-Atb2 (Alpha tubulin 2), which labels microtubules and hence served as a cell cycle stage marker. In interphase cells, Sts5 had a mostly diffuse cytoplasmic localization, however during mitosis it appeared to localize in dotted, cytoplasmic bodies (Figure 5A). The number of Sts5 dots increased throughout mitosis and peaked coinciding with the assembly of the Post Anaphase Array (PPA) of microtubules (Figure 5B). Time-lapse movies of mitotic cells also confirmed that the number of cytoplasmic dots increased until the formation of the PAA and sharply dropped to zero as cells entered interphase (Figure S6). Previous studies of Sts5 [69] showed that it was required for correct cell growth and actin patch localization during interphase. Taken together with our results, this suggests that the cell cycle controls Sts5 activity by gradually sequestering it in cytoplasmic bodies during mitosis.

## Discussion

In this work, we have carried out the first network analysis based, large-scale identification of proteins linking various cellular processes in the fission yeast protein-protein interaction network. Although data for fission yeast mostly comes from manually annotated experiments, literature mining and computational inference, the network displays features comparable to those observed in other organisms. We have shown that the relationship between lethality and different network measures holds in fission yeast, and that network based approaches can give meaningful and interesting results even in organisms lacking high-throughput interaction experiments.

Our analysis of the core network of all proteins regulating cell cycle, cytokinesis, and polarized growth revealed a striking degree of functional modularity, which we have found to be highly robust to the deletion of key nodes in the network. This functional modularity was also observed when examining the communities detected by a clique propagation algorithm. Detected communities had very low heterogeneity between the functional annotations of member proteins. We investigated this modularity further by using a network approach to identify linker proteins bridging different functional categories. We propose a new network measure, linkerity, which is the ratio of the ranking by betweennness centrality measures of all the nodes belonging to a given sub-network considered in the sub-network alone and considered in the context of a larger network (Figure 4A). This new network measure does not appear to show strong correlation with other existing network measures (Text S3). Due to the non-linear distribution of betweenness centrality measures in real systems [48], it might be necessary to normalize this linkerity measure in case linkers between large sub-networks are investigated.

We tested this concept on the connections of the polarized cell growth regulatory network to the cytokinesis and cell cycle networks of fission yeast cells. These are highly characterized and strongly interacting networks and the connection between these processes is of high importance in other organisms [7], [13], [80]–[82]. We confirmed that many of the highest linkerity scoring proteins in the polarity network were already known to play important roles in multiple processes. Among these the F-BAR protein Cdc15 provide good validation as it was already shown to play a role in switching from polarized growth to cytokinetic-actin ring formation in mitosis [83]. Similarly Skb1 [84] and Cdr1 [17], [23] were shown to serve as links between cell cycle and cell polarity. All these proteins shifted from a low ranking in the polarity network to a high rank in the core network (Table 2), and thus their role in polarity regulation might come from the pleotropic behavior of these proteins or from their active role in connecting polarized growth regulation to cell cycle and cytokinesis. We also discovered that the proteins with high linkerity tend to interact with a more diverse set of proteins than those with low linkerity. This suggests that high linkerity proteins might play a pleiotropic role by linking together different functional processes [85], [86].

Sts5 had the second highest ranking in the polarity network among the top ten linkerity proteins (after actin, Act1 that is also essential for cytokinesis). Sts5 is known to play an important role in controlling the localization of the actin machinery to cell ends during interphase, although Sts5 is localized in the cytoplasm [69]. We have shown that Sts5 is localized in cytoplasmic dots during mitosis, but diffuse during interphase, implying that its localization is cell cycle regulated. Growing tip localized polarity proteins change their localization when cells enter mitosis [13], [87], but it is not expected from a cytoplasmic protein to localize into clusters in a cell cycle dependent manner. The overall level of Sts5 protein slightly increases upon entry to mitosis (Figure S6), but its activity reaches its lowest level as its accumulation into cytoplasmic dots reaches a peak. This suggests that the cell cycle controls polarity by sequestering Sts5 in and out of cytoplasmic bodies, and the triggered release and sequestration function as switches between polarized cell growth and cytokinesis. The exact nature of those cytoplasmic bodies is still unclear, however the budding yeast Sts5 homologue SSD1 was shown to localize to P-bodies [88], the cytoplasmic centers of mRNA degradation. Interestingly, like Sts5, Ssp2 and the stress pathway kinase Wis4 are also localized into cytoplasmic dots [89] and it was proposed that the stress pathway and Sts5 might act in opposing manner on cell polarity [68]. It will be important in the future to investigate if these proteins co-localize in the observed cytoplasmic dots and how these are exactly controlled by the cell cycle.

Sts5 was previously shown to genetically interact with members of the stress pathway [69]. A number of other kinases associated with stress response (such as Sty1, Skb1, Orb6, Pmk1, Mkh1) have been shown to have defects in NETO [84], [89] and many of these appear highly ranked in our linkerity lists (Table 2). Furthermore, the cell end-localized polarity factor Tea4 was also shown to interact with the stress pathway [90]. These make the stress pathway a particularly intriguing target for further analysis in the search for proteins linking cell cycle and polarity, as it may play a special role as a pleiotropy integrator of both internal and external cellular signals in response to different stimuli in fission yeast and also in higher eukaryotes [91], [92]. The linkerity analysis of cytokinesis and cell cycle regulatory proteins (bottom parts of Table 2) also give some interesting predictions. For instance the high linkerity of the transcription factor Cdc10 [93] in the cell cycle network suggests its role controlling the transcription of important polarity and cytokinesis genes, especially with key regulators, such as Cdc15, Scd2, Sts5, Rho4 and Sid2 having periodic transcriptional profile [74], [79].

While we believe that the method presented here can be applied to other organisms and cellular processes to find linker proteins, different model organisms offer unique advantages and challenges. In this study, we took advantage of the extensive annotation of proteins by the fission yeast community to define discrete sub-networks, bypassing the very difficult problems involved in defining meaningful ‘communities’ using purely network based approaches [46], [54], [94]. While this approach has its advantages, it is important to be aware of any partial overlaps between the used GO terms due to the presence of common child terms. The amount of overlap between child terms is also not consistent across multiple organisms, requiring special care when doing comparisons that involve multiple organisms (for example, the “regulation of cell cycle cytokinesis” is a child term of both “regulation of cell cycle” and “cytokinesis” and it contains 47 proteins in fission yeast, and only 4 proteins in budding yeast). Furthermore, while we have shown that the ranking of proteins within the communities is robust to noise, the actual communities detected by various algorithms as well as the structure of the network are strongly influenced by the granularity and quality of the interaction data used (Text S4 and [95]). In fission yeast, where interaction data is relatively sparse but there is extensive functional annotation, it makes sense to use GO annotations to define functional sub-networks [38]. Very recent network predictions based on machine-learning methods [33] will enable us to perform more careful analysis in this organism as well. Other organisms with larger gene sets will often have a lower annotation coverage [96]; in these cases functional groups in the PPI network need to be identified by community detection algorithms or predefined by the authors [80]. Once such functional groups are established, the described method provides a good means to identify proteins likely to have a role in connecting functional regulatory networks in any organism. Likewise, the defined linkerity measure can be used to identify key linker nodes of sub-networks in any complex network [54], [97]–[99].

## Materials and Methods

### Bioinformatics data compilation

To obtain a list of proteins associated with specific cellular processes, we used the Gene Ontology (http://www.geneontology.org/) and downloaded all gene products associated with a given term. It is important to note that while ‘cytokinesis’ (GO:0000910) and ‘cell cycle regulation’ (GO:0051726) have specific terms that cover all proteins commonly associated with those processes, for polarity *S. pombe* proteins are split between ‘establishment or maintenance of cell polarity’ (GO:0007163) and ‘cell morphogenesis’ (GO:0000902). In the analysis, we thus used the umbrella term ‘polarity’ to include proteins in both of these categories. Data in STRING (http://string-db.org/) is present at different confidence scores. Confidence scores in STRING represent the likelihood of the two proteins actually interacting, and depend on the reliability of the source of the interaction. For example, an interaction that is reported in a single experiment will have a far higher confidence score than an interaction that is inferred through text mining or homology alone. We studied the effect of a cutoff in this confidence score on network size defined as the fraction of all proteins connected with at least one other protein; the main component size defined as the fraction of all proteins connected to the largest component in the network; and the edge fraction defined as the fraction of all edges found, compared to the theoretical maximum. To download the number of PubMed abstracts mentioning the name of a protein in the network, we relied on the Entrez module of the Biopython package (http://biopython.org/wiki/Biopython). Statistical analysis, including calculation of correlations, was carried out using the Statistics module of the SciPy package (http://www.scipy.org/). All network measures were calculated using pre-existing algorithms implemented in NetworkX (http://networkx.lanl.gov/). For community structure detection we used the k-clique propagation algorithm originally described in [53], and implemented in NetworkX [100]. Packages were packaged in the Enthought Python Distribution courtesy of Enthought (http://www.enthought.com/).

### Network analysis workflow

The pipeline used to create the networks was:

1. We connected to the MySQL Gene Ontology database using custom python scripts, and downloaded all proteins associated with a given biological process.
2. We took all proteins downloaded and used them to query STRING, downloading all the information about protein-protein interactions in PSI-MI-TAB format. It is important to note that STRING and Gene Ontology sometimes identify the same gene by a different name, therefore special care was taken to use consistent nomenclature.
3. We parsed the PSI-MI-TAB file and transformed it into a NetworkX graph, which we could then study using both algorithms built into NetworkX as well as custom scripts.

We repeated the analysis described in the main text using networks obtained from BioGRID. In that case, instead of using STRING in step 2 we parsed the full network of a given organism from a PSI-MI-TAB file available for download on the BioGRID website, then extracted the sub-graph containing the nodes obtained in step 1 and edges of physical interactions stored in the database. The results presented are based on the state of all databases on 13 March 2012. The calculated network measures, PubMed citations and all presented numerical results are detailed in the Excel files of Tables S1, S2, S3, S4.

All Python scripts used to download data from databases as well as for analysis are available upon request.

### Strains and strain construction

The *S. pombe* strain used in this study was MH123 (*h- sts5-3GFP-L-nat Z2-mCh-atb2-hph leu1 ura4 ade6-M216 his7*). Conventional PCR-based gene targeting methods for *S. pombe* were used for gene tagging [101]–[103].

### Live microscopy cell imaging

Prior to imaging, *S. pombe* strains were grown at 32°C in yeast extract with supplements (YES) (*5*) to exponential growth. Aliquots of 300 ml cells were mounted onto 1.5 coverslip glass-bottomed plastic dishes (MatTek; P35G-1.5-14-C) pre-coated with 10 ml 1 mg/ml lectin (Sigma; L1395 and Patricell Ltd; L-1301-25) that had been allowed to air dry. After a 30-minute incubation, cells unbound to the lectin-coated glass were removed by washing with minimal medium (EMM) [101]–[103] and the bound cells were kept in a final suspension of 1 ml EMM.

Imaging was performed with both: an OMX microscope (Applied Precision) in conventional resolution mode, with an Olympus UPlanSapo ×100 oil immersion lens (NA1.4) and 1.512 RI immersion oil (Applied Precision); and a DeltaVision microscope (Applied Precision), comprising an Olympus 1×71 widefield microscope, an Olympus UPlanSapo ×100 oil immersion lens (NA1.4) and an Photometrics CoolSNAP HQ^2^ camera. For analysis of Sts5-3GFP speckle number, stacks were taken at 0.4 um apart for 16 focal planes on the Deltavision microscope. Time lapses were taken for single focal planes at ten-minute intervals on the DeltaVision microscope.

### Automated analysis of Sts5-3GFP speckle number

Cells within microscopy image fields were automatically segmented from the transmitted light channel using an algorithm developed in-house and coded in Matlab. For each cell, the cell-cycle stage was determined manually by looking at the mCh-Atb2 channel.Sts5-GFP speckles were detected using the spot detection module of the ICY software (http://icy.bioimageanalysis.org/; [104]

## Supporting Information

Figure S1 **The cell cycle + cytokinesis + polarity = core interaction network of budding yeast proteins.** (**A**) Venn diagram showing the overlap among the different Gene Ontology functional groups in the proteins present in the core network of budding yeast. Proteins with multiple functional annotations have colours that are the sum of the colours of the individual functional annotations, proteins belonging to all three functional groups are in white. (**B**) Protein-protein interaction in the budding yeast core network (from the STRING database at cutoff 0.7). Node colour same as in panel A. Node size is proportional to degree of the protein, and node order within a category (clockwise) is also determined by degree. 469 Black edges link proteins that do not share functional annotations, while 2146 grey edges link proteins that have at least one common GO annotation (thus white nodes have only grey links). White nodes (nodes belonging to all categories) are shown in the inner circle in the middle of the network.
(PDF)

Figure S2 **Scale free distribution of networks.** We calculated the degree of every node in the largest connected component of the genomwide network for both fission yeast (**A**) and budding yeast (**B**). We then calculated a histogram for frequency of degree (with number of bins equal to the maximum degree observed in the network) and plotted log(frequency) vs log(degree). Best fits to log(*P*(*k*))∼log (*ck^−γ^*) were calculated using a least square minimization algorithm from scipy (http://www.scipy.org/).
(PDF)

Figure S3 **Functional modularity in the core networks.** To calculate how much the functional modularity (the ratio of interactions between nodes with a shared GO category versus interactions between nodes with no GO category in common) observed for the core network of budding and fission yeast deviated from a random network, we kept all the category labels for all the nodes, but rewired the network either completely at random (**A**, **C**), or using a method that preserves degree-distribution (**B**, **D**) [108]. To rewire the networks at random, we removed every edge from the network then added an edge between any two nodes chosen at random until the total amount of edges in the network was equal to the original amount. To preserve degree distribution of the networks, we performed a double edge swap across the network. We picked two existing edges at random between nodes (**u,v**) and (**x,y**). We then added an edge between (**u,x**) and (**y,v**) and removed the original edge. Red arrows indicate the observed ratio for the core network, the distributions represent 1000 different random networks and their functional modularity.
(PDF)

Figure S4 **Robustness analysis for the betweenness centrality ranking for polarity, cytokinesis and cell cycle networks in fission yeast.** We analysed the robustness of ranking proteins by BC centrality in the presence of imperfect network interaction data. We added 10% extra edges at random to the network, calculated BC for every node after adding the edges, and ranked all the proteins. We calculated the mean and standard deviation for the rank of every protein in the network after repeating the procedure 1000 times. We normalized the rank of all proteins (Rank/number of nodes) and plotted the top 20% of nodes and their mean and standard deviation. The blue dotted line represents the cutoff for top 10% nodes, and the red dotted line represents the cutoff for top 20% of nodes. **A**, **B**, **C** are the top 20% proteins of regulation of cell cycle, cytokinesis and polarity of fission yeast.
(PDF)

Figure S5 **Robustness analysis of linkerity of proteins in the fission yeast polarity network.** We systematically analysed the robustness of linkerity in the presence of imperfect network interaction data. We added 10% edges preferentially to nodes with high degree (**A**) or removed 10% edges at random (**B**) to the core network. In the preferential attachment model, the probability P that a given node N had of gaining an edge was directly proportional to its degree *P(N)*∼*Degree(N)*. In the random model *P(N)*∼*k* where *k* is a constant. Probabilities were normalized to increase or decrease the total edges of the network by 10%. We calculated the mean and standard deviation for the betweenness centrality of every protein belonging to the polarity sub-network after repeating the procedure 1000 times. We plotted the top 20% of nodes and their mean and standard deviation. The blue dotted line represents the cutoff for top 10% nodes, and the red dotted line represents the cutoff for top 20% of nodes.
(PDF)

Figure S6 **Time-lapse analysis of Sts5 localization in fission yeast cells.** Microtubules are visualized using mCherry labeled tubulin (Atb2) to identify cell cycle stage (**A** and **B** right column and Sts5-3GFP is visualized on the left). As the cell cycle progresses, Sts5 starts to accumulate into cytoplasmic dots, which then rapidly disappear upon septum formation. **C** is an automatic quantification of the amount of cytoplasmic dots in cells at different stages of the cell cycle.
(PDF)

Table S1 **Analysis of the genome-wide fission yeast network.** See detailed description under Table S2.
(XLS)

Table S2 **Analysis of the genome-wide budding yeast network.** Tabulated file (in .xls format) containing network measures for all protein in the largest connected component of the genome-wide network of fission (S1) and budding (S2) yeast. Columns include: **Common name:** Common name. **Systematic name:** Systematic name (for fission yeast), GO database ID (for budding yeast) **Description:** Brief description of known protein activity. **PubMed count:** Number of abstracts discussing that particular protein in fission yeast available in PubMed. **Lethality: E** (Essential) If deletion of the gene causes lethality, **V** (Viable) otherwise. **Scores**: Betweenness Centrality and Degree scores for the protein in the network using either STRING interaction data (at increasing cutoffs, 0.4, 0.7, 0.9) or data from BioGRID (only the physical protein-protein interaction data). Genes with no entry at a given cutoff have no other interactions with any proteins in the network.
(XLS)

Table S3 **Analysis of the core fission yeast network.** See detailed description under Table S4.
(XLS)

Table S4 **Analysis of the core budding yeast network.** Tabulated file (in .xls format) containing network measures for all protein in the core network of fission (S3) and budding (S4) yeast. Columns include: **Common name:** Common name. **Systematic name:** Systematic name (for fission yeast), GO database ID (for budding yeast) **Description:** Brief description of known protein activity. **PubMed count:** Number of abstracts discussing that particular protein in fission yeast available in PubMed. **Lethality: E** (Essential) If deletion of the gene causes lethality, **V** (Viable) otherwise. **GO Categories:** Which of the three categories (Cytokinesis (**CY**), Polarity (**P**), Cell Cycle (**CC**)) does the protein belong too? **Scores**: Betweenness Centrality and Degree scores and ranks for all the sub-networks the protein belongs to, as well as the core network. In the sub-network, betweenness rank (*Rank_sub-network_*) is calculated by ranking all the proteins from highest to lowest according to their betweenness. In the core network, the betweenness rank (*Rank_core_*) is calculated only between proteins that are found in the original sub-network. To avoid artifacts due to the presence of multiple proteins with 0 betweenness, we assign consecutive proteins with the exact same score have the same rank, which is simply defined as the average of their ranking [109]. For example: in a network of 6 proteins [**A**, **B**, **C**, **D**, **E**, **F**], with BC values of [10, 10, 7, 5, 5, 5], the ranking would be: [(**A**, 1.5), (**B**, 1.5), (**C**, 3), (**D**, 5), (**E**, 5), (**F**, 5)] **Linkerity**: Linkerity calculated for all the categories as given in Equation 1. Note that the linkerity for a protein that doesn't shift in rank is 1 by definition.
(XLS)

Table S5 **All members of the cliques identified on** **Figure 3B** **.** List of all proteins belonging to the cliques described in Figure 3. Clique 1 corresponds to the top left clique in Figure 3B, with cliques increasing moving from left to right.
(PDF)

Text S1 **Predicting essentiality by network measures in fission and budding yeast.**
(PDF)

Text S2 **Analysis of the neighbors of high linkerity proteins.**
(PDF)

Text S3 **Correlation between linkerity and other network measures.**
(PDF)

Text S4 **Linkerity at various network confidences.**
(PDF)
