# Supplementary material for: Linkers of Cell Polarity and Cell Cycle Regulation in the Fission Yeast Protein Interaction Network
Source: PLoS Comput Biol. 2012 Oct 18;8(10):e1002732. doi: 10.1371/journal.pcbi.1002732 (PMC3475659; doi:10.1371/journal.pcbi.1002732)
Supplement: Figure S1 — The cell cycle + cytokinesis + polarity = core interaction network of budding yeast proteins. (A) Venn diagram showing the overlap among the different Gene Ontology functional groups in the proteins present in the core network of budding yeast. Proteins with multiple functional annotations have colours that are the sum of the colours of the individual functional annotations, proteins belonging to all three functional groups are in white. (B) Protein-protein interaction in the budding yeast core network (from the STRING database at cutoff 0.7). Node colour same as in panel A. Node size is proportional to degree of the protein, and node order within a category (clockwise) is also determined by degree. 469 Black edges link proteins that do not share functional annotations, while 2146 grey edges link proteins that have at least one common GO annotation (thus white nodes have only grey links). White nodes (nodes belonging to all categories) are shown in the inner circle in the middle of the network. (PDF) [file pcbi.1002732.s001.pdf]

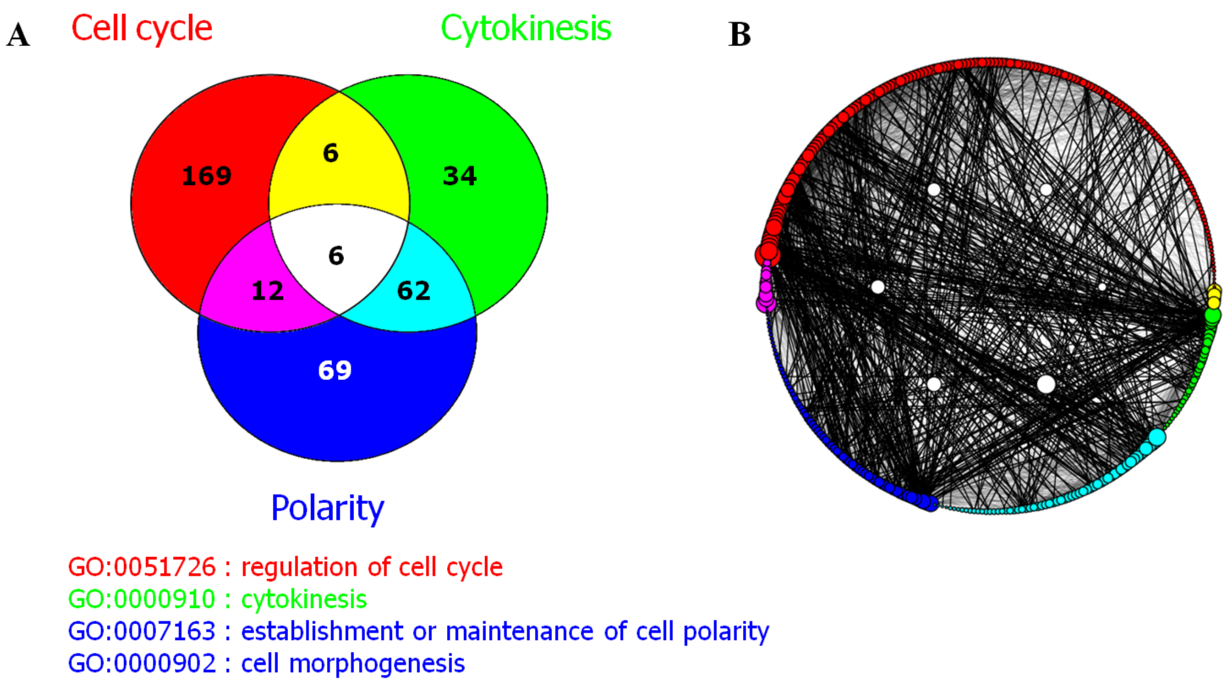

Figure S1: The cell cycle + cytokinesis + polarity = core interaction network of budding yeast proteins (**A**) Venn diagram showing the overlap among the different Gene Ontology functional groups in the proteins present in the core network of budding yeast. Proteins with multiple functional annotations have colours that are the sum of the colours of the individual functional annotations, proteins belonging to all three functional groups are in white. (**B**) Protein-protein interaction in the budding yeast core network (from the STRING database at cutoff 0.7). Node colour same as in panel A. Node size is proportional to degree of the protein, and node order within a category (clockwise) is also determined by degree. 469 Black edges link proteins that do not share functional annotations, while 2146 grey edges link proteins that have at least one common GO annotation (thus white nodes have only grey links). White nodes (nodes belonging to all categories) are shown in the inner circle in the middle of the network.
