# Supplementary material for: Linkers of Cell Polarity and Cell Cycle Regulation in the Fission Yeast Protein Interaction Network
Source: PLoS Comput Biol. 2012 Oct 18;8(10):e1002732. doi: 10.1371/journal.pcbi.1002732 (PMC3475659; doi:10.1371/journal.pcbi.1002732)
Supplement: Figure S2 — Scale free distribution of networks. We calculated the degree of every node in the largest connected component of the genomwide network for both fission yeast (A) and budding yeast (B). We then calculated a histogram for frequency of degree (with number of bins equal to the maximum degree observed in the network) and plotted log(frequency) vs log(degree). Best fits to log(P(k))∼log (ck−γ) were calculated using a least square minimization algorithm from scipy (http://www.scipy.org/). (PDF) [file pcbi.1002732.s002.pdf]

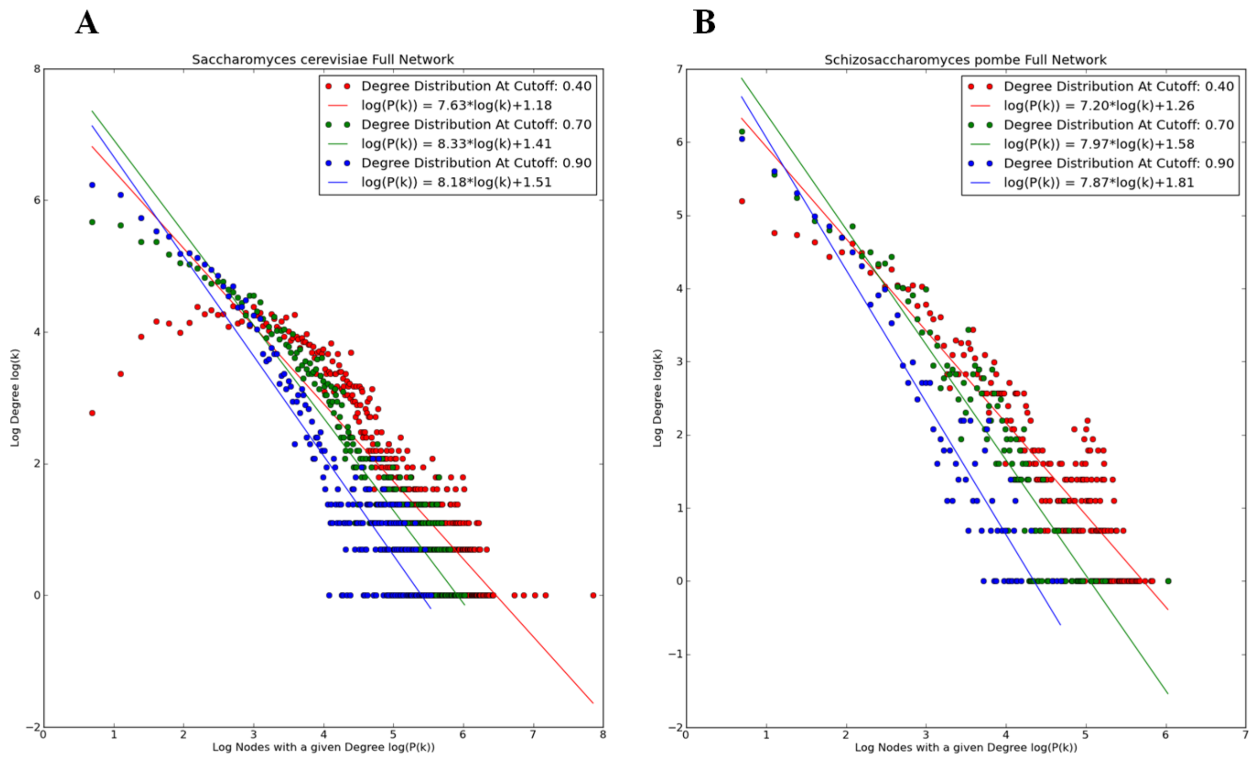


**Figure S2: Scale Free Distribution of Networks.** We calculated the degree of every node in the largest connected component of the genomwide network for both fission yeast (**A**) and budding yeast (**B**). We then calculated a histogram for frequency of degree (with number of bins equal to the maximum degree observed in the network) and plotted log(frequency) vs log(degree). Best fits to $\log(P(k)) \sim \log(ck^{-\gamma})$ were calculated using a least square minimization algorithm from scipy (http://www.scipy.org/).
