# Supplementary material for: Linkers of Cell Polarity and Cell Cycle Regulation in the Fission Yeast Protein Interaction Network
Source: PLoS Comput Biol. 2012 Oct 18;8(10):e1002732. doi: 10.1371/journal.pcbi.1002732 (PMC3475659; doi:10.1371/journal.pcbi.1002732)
Supplement: Figure S3 — Functional modularity in the core networks. To calculate how much the functional modularity (the ratio of interactions between nodes with a shared GO category versus interactions between nodes with no GO category in common) observed for the core network of budding and fission yeast deviated from a random network, we kept all the category labels for all the nodes, but rewired the network either completely at random (A, C), or using a method that preserves degree-distribution (B, D) [108]. To rewire the networks at random, we removed every edge from the network then added an edge between any two nodes chosen at random until the total amount of edges in the network was equal to the original amount. To preserve degree distribution of the networks, we performed a double edge swap across the network. We picked two existing edges at random between nodes (u,v) and (x,y). We then added an edge between (u,x) and (y,v) and removed the original edge. Red arrows indicate the observed ratio for the core network, the distributions represent 1000 different random networks and their functional modularity. (PDF) [file pcbi.1002732.s003.pdf]

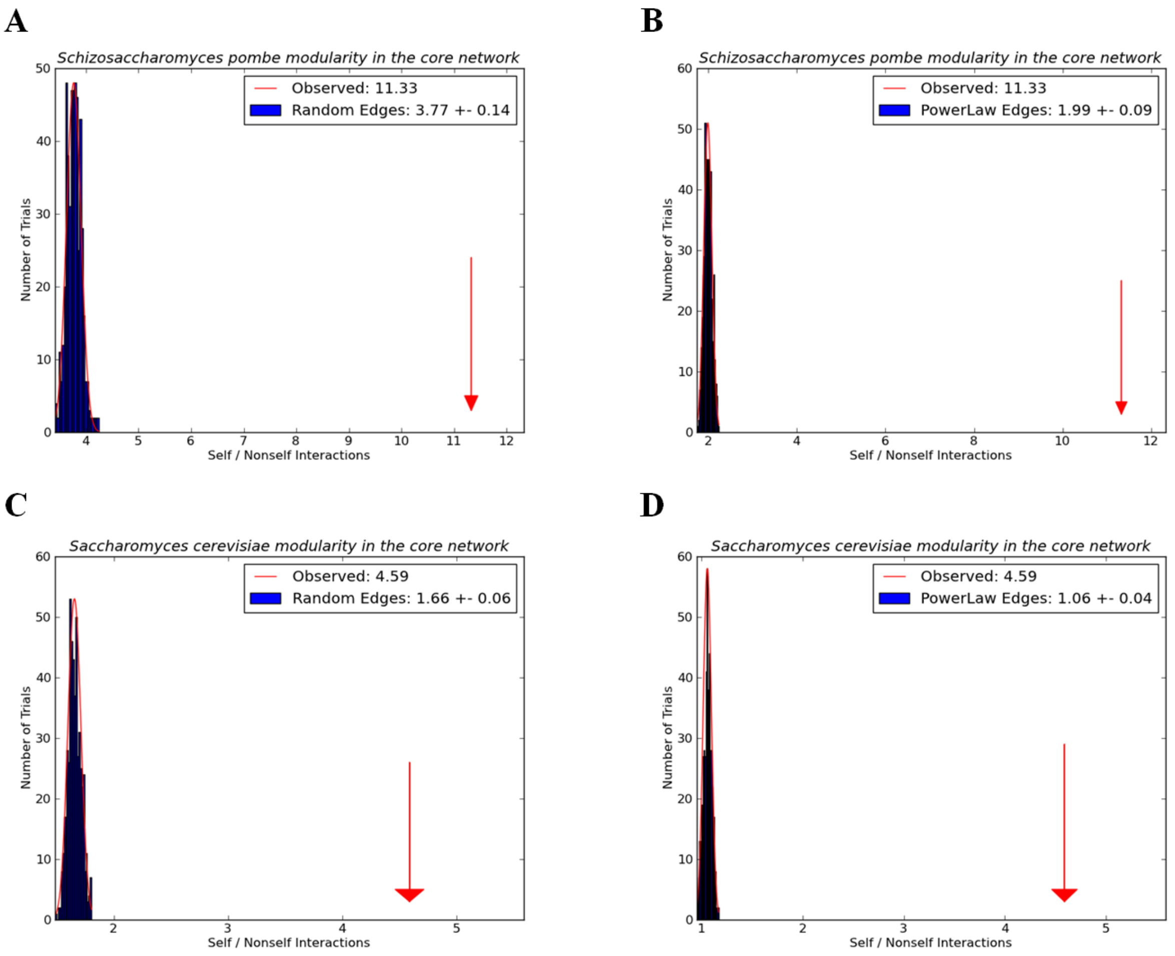

Figure S3: Functional modularity in the core networks. To calculate how much the functional modularity (the ratio of interactions between nodes with a shared GO category versus interactions between nodes with no GO category in common) observed for the core network of budding and fission yeast deviated from a random network, we kept all the category labels for all the nodes, but rewired the network either completely at random (**A**, **C**), or using a method that preserves degree-distribution (**B**, **D**) (Gkantsidis *et al*, 2003). To rewire the networks at random, we removed every edge from the network then added an edge between any two nodes chosen at random until the total amount of edges in the network was equal to the original amount. To preserve degree distribution of the networks, we performed a double edge swap across the network. We picked two existing edges at random between nodes (**u,v**) and (**x,y**). We then added an edge between (**u,x**) and (**y,v**) and removed the original edge.. Red arrows indicate the observed ratio for the core network, the distributions represent 1000 different random networks and their functional modularity.
