# Supplementary material for: Linkers of Cell Polarity and Cell Cycle Regulation in the Fission Yeast Protein Interaction Network
Source: PLoS Comput Biol. 2012 Oct 18;8(10):e1002732. doi: 10.1371/journal.pcbi.1002732 (PMC3475659; doi:10.1371/journal.pcbi.1002732)
Supplement: Figure S4 — Robustness analysis for the betweenness centrality ranking for polarity, cytokinesis and cell cycle networks in fission yeast. We analysed the robustness of ranking proteins by BC centrality in the presence of imperfect network interaction data. We added 10% extra edges at random to the network, calculated BC for every node after adding the edges, and ranked all the proteins. We calculated the mean and standard deviation for the rank of every protein in the network after repeating the procedure 1000 times. We normalized the rank of all proteins (Rank/number of nodes) and plotted the top 20% of nodes and their mean and standard deviation. The blue dotted line represents the cutoff for top 10% nodes, and the red dotted line represents the cutoff for top 20% of nodes. A, B, C are the top 20% proteins of regulation of cell cycle, cytokinesis and polarity of fission yeast. (PDF) [file pcbi.1002732.s004.pdf]

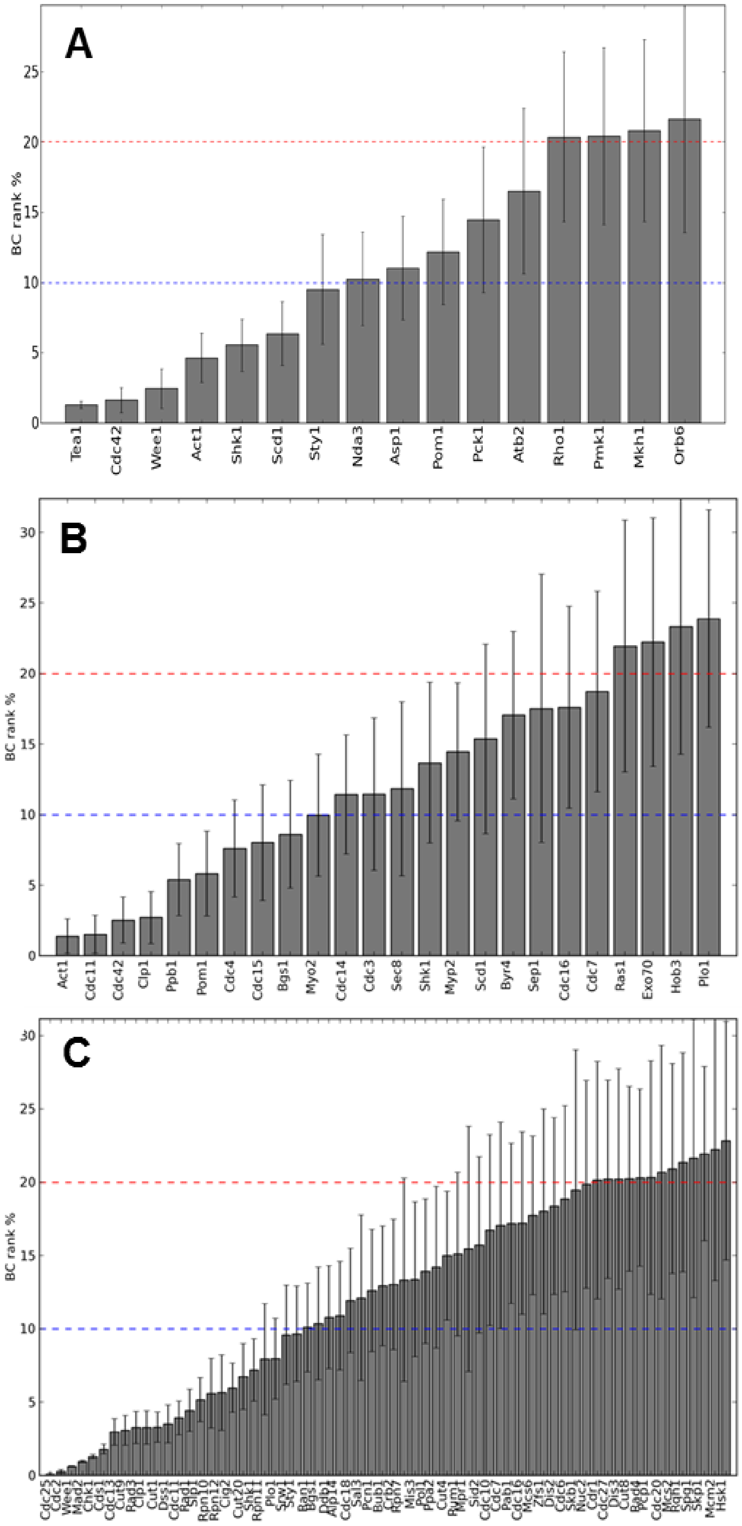

Figure S4: Robustness analysis for the betweenness centrality ranking for polarity, cytokinesis and cell cycle networks in fission yeast. We analysed the robustness of ranking

proteins by BC centrality in the presence of imperfect network interaction data. We added 10% extra edges at random to the network, calculated BC for every node after adding the edges, and ranked all the proteins. We calculated the mean and standard deviation for the rank of every protein in the network after repeating the procedure 1000 times. We normalized the rank of all proteins (Rank / number of nodes) and plotted the top 20% of nodes and their mean and standard deviation. The blue dotted line represents the cutoff for top 10% nodes, and the red dotted line represents the cutoff for top 20% of nodes. **A**, **B**, **C** are the top 20% proteins of regulation of cell cycle, cytokinesis and polarity of fission yeast.
