# Supplementary material for: Linkers of Cell Polarity and Cell Cycle Regulation in the Fission Yeast Protein Interaction Network
Source: PLoS Comput Biol. 2012 Oct 18;8(10):e1002732. doi: 10.1371/journal.pcbi.1002732 (PMC3475659; doi:10.1371/journal.pcbi.1002732)
Supplement: Figure S5 — Robustness analysis of linkerity of proteins in the fission yeast polarity network. We systematically analysed the robustness of linkerity in the presence of imperfect network interaction data. We added 10% edges preferentially to nodes with high degree (A) or removed 10% edges at random (B) to the core network. In the preferential attachment model, the probability P that a given node N had of gaining an edge was directly proportional to its degree P(N)∼Degree(N). In the random model P(N)∼k where k is a constant. Probabilities were normalized to increase or decrease the total edges of the network by 10%. We calculated the mean and standard deviation for the betweenness centrality of every protein belonging to the polarity sub-network after repeating the procedure 1000 times. We plotted the top 20% of nodes and their mean and standard deviation. The blue dotted line represents the cutoff for top 10% nodes, and the red dotted line represents the cutoff for top 20% of nodes. (PDF) [file pcbi.1002732.s005.pdf]

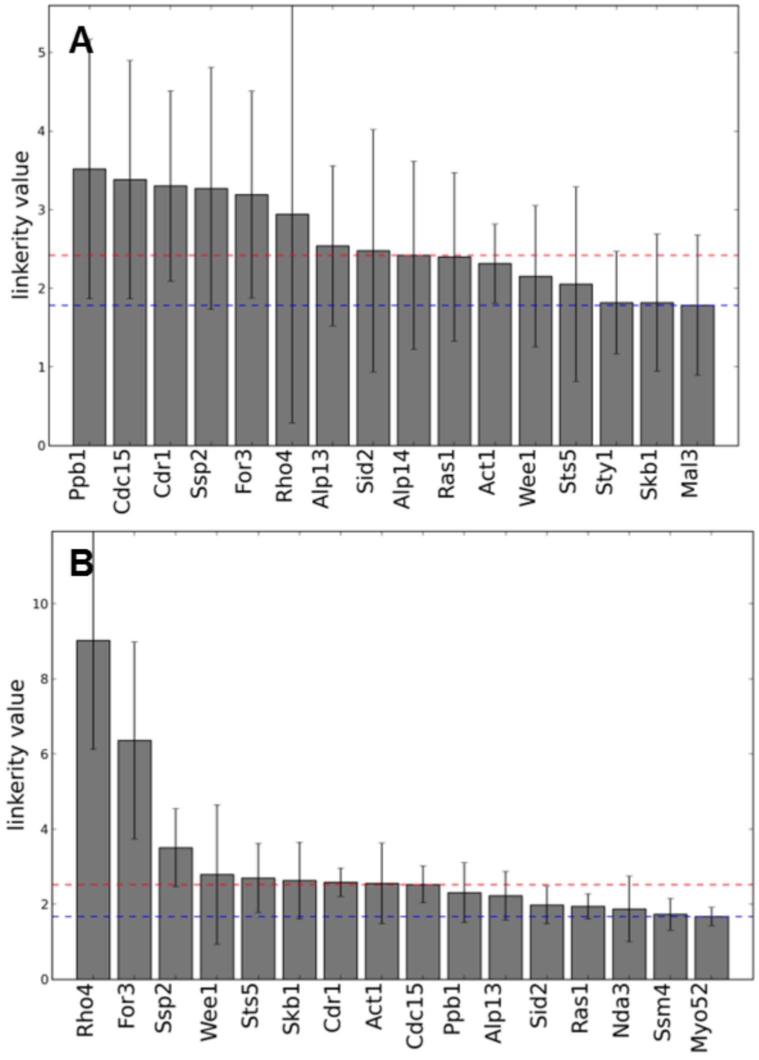

Figure S5: Robustness analysis of linkerity of proteins in the fission yeast polarity network. We systematically analysed the robustness of linkerity in the presence of imperfect network interaction data. We added 10% edges preferentially to nodes with high degree (**A**) or removed 10% edges at random (**B**) to the core network. In the preferential attachment model, the probability P that a given node N had of gaining an edge was directly proportional to its degree *P(N) ~ Degree(N).* In the random model *P(N) ~ k* where *k* is a constant. Probabilities were normalized to increase or decrease the total edges of the network by 10%. We calculated the mean and standard deviation for the betweenness centrality of every protein belonging to the polarity subnetwork after repeating the procedure 1000 times. We plotted the top 20% of nodes and their mean and standard deviation. The blue dotted line represents the cutoff for top 10% nodes, and the red dotted line represents the cutoff for top 20% of nodes.
