# Supplementary material for: Linkers of Cell Polarity and Cell Cycle Regulation in the Fission Yeast Protein Interaction Network
Source: PLoS Comput Biol. 2012 Oct 18;8(10):e1002732. doi: 10.1371/journal.pcbi.1002732 (PMC3475659; doi:10.1371/journal.pcbi.1002732)
Supplement: Figure S6 — Time-lapse analysis of Sts5 localization in fission yeast cells. Microtubules are visualized using mCherry labeled tubulin (Atb2) to identify cell cycle stage (A and B right column and Sts5-3GFP is visualized on the left). As the cell cycle progresses, Sts5 starts to accumulate into cytoplasmic dots, which then rapidly disappear upon septum formation. C is an automatic quantification of the amount of cytoplasmic dots in cells at different stages of the cell cycle. (PDF) [file pcbi.1002732.s006.pdf]

A

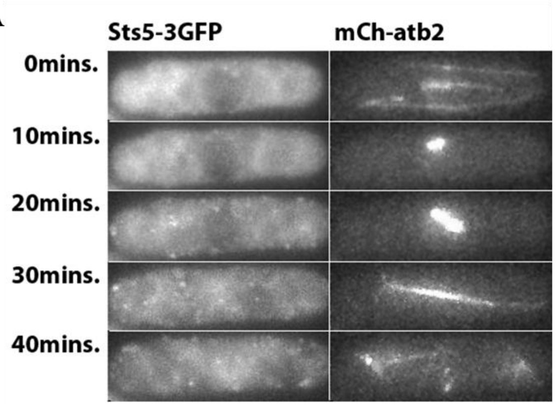

B

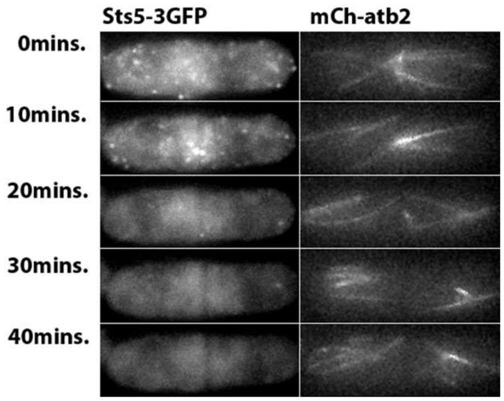

C

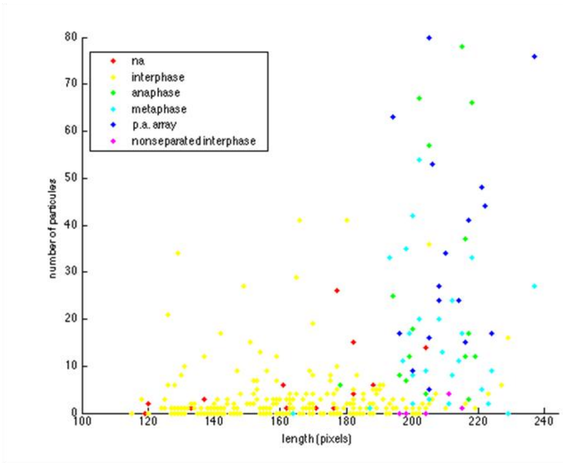

Figure S6: Time-lapse analysis of Sts5 localization in fission yeast cells. Microtubules are visualized using mCherry labeled tubulin (Atb2) to identify cell cycle stage (**A** and **B** right column and Sts5-3GFP is visualized on the left). As the cell cycle progresses, Sts5 starts to accumulate into cytoplasmic dots, which then rapidly disappear upon septum formation.

C is an automatic quantification of the amount of cytoplasmic dots in cells at different stages of the cell cycle.
