# Supplementary material for: Linkers of Cell Polarity and Cell Cycle Regulation in the Fission Yeast Protein Interaction Network
Source: PLoS Comput Biol. 2012 Oct 18;8(10):e1002732. doi: 10.1371/journal.pcbi.1002732 (PMC3475659; doi:10.1371/journal.pcbi.1002732)
Supplement: Table S5 — All members of the cliques identified on Figure 3B . List of all proteins belonging to the cliques described in Figure 3. Clique 1 corresponds to the top left clique in Figure 3B, with cliques increasing moving from left to right. (PDF) [file pcbi.1002732.s011.pdf]

Table S5: All members of the cliques identified on Figure 3B

List of all proteins belonging to the cliques described in Figure 3. Clique 1 corresponds to the top left clique in Figure 3B, with cliques increasing moving from left to right.

Clique 1:

cdc20, rhp18, ned8, srk1, apc13, mcs6, cut9, ppa2, rem1, cdr2, pcn1, mus81, mad2, cdc17, srw1, mis16, rad24, slp1, cdc2, sty1, pkl1, dam1, skp1, mik1, mcs2, mid1, cdc23, cdc22, cut23, rad25, cdc13, hcn1, dfp1, rqh1, cdc18, ddb1, chk1, klp5, rad9, spp1, suc1, mcm2, swi1, mis3, cdc25, rpc40, apc2, cds1, mrc1, slm9, hus1, cut8, wis1, rad1, apc14, nuc2, pyp2, pof1, cdc1, bir1, cul1, taz1, pop1, crb2, alp7, bgs1, sep1, rad26, ark1, apc15, nda3, cut12, cdc21, apc11, rfc2, gtb1, pyp1, cut20, shk1, cdc11, bub1, wos2, sts5, apc10, fin1, cdc24, pyk1, cut1, bub3, cdc10, pub1, myh1, mcs4, puc1, cut2, msh2, pol1, rpb4, alp14, pop2, tel1, rum1, pka1, blt1, fcp1, taf73, rfc3, win1, cmk2, rad3, hsk1, klp2, rad17, cdc27, mad1, cdc6, cdr1, klp6, mad3, orc1, wee1, pic1, apc5, mcm6, rad4, cig2, cut4, mcl1, nuf2, spp2, rhp23, skb1, pmh1, clp1, atb2, cdc37, set1, cig1, mal3, pfh1, cdt2

Clique 2:

pre6, rpt2, rpn11, SPAC13C5.01c, ecm29, rpn6, SPAC31A2.04c, pup1, rhp23, pup2, rpn501, SPAC6G10.04c, SPBC646.16, rpt6, pts1, rpn7, pam1, rpn9, rpt4, SPBC577.10, rpn3, rpt5, rpn10, mts4, rpn8, rpn12, pre10, pre3, cut8, pre8, dss1, rpn502, rpn2, rpt1

Clique 3:

cdc15, clp1, sid1, cdc11, par1, dma1, ppb1, cdc7, scw1, mob1, pab1, lsk1, myp2, sid2, myo2, spg1, sid4, cdc14, zfs1, plo1, bgs1, cdc16, byr4

Clique 4:

scd1, shk1, ras1, rho1, scd2, tea1, gef1, klp6, cdc42, klp5

Clique 5:

rho1, rgf3, rgf1, pck2, rga1, cdc42, pck1

Clique 6:

act1, stg1, acp1, adf1, fim1, rng3, acp2

Clique 7:

tip1, atb2, mad2, cdc16, mal3, ssm4, tea2

Clique 8:

spd1, pcu4, csn1, ddb1, nep1, cdt2

Clique 9:

gtb1, alp6, gfh1, pcp1, alp4, mad2

Clique 10:

win1, byr2, mkh1, wis4, wis1
