# Supplementary material for: Linkers of Cell Polarity and Cell Cycle Regulation in the Fission Yeast Protein Interaction Network
Source: PLoS Comput Biol. 2012 Oct 18;8(10):e1002732. doi: 10.1371/journal.pcbi.1002732 (PMC3475659; doi:10.1371/journal.pcbi.1002732)
Supplement: Text S1 — Predicting essentiality by network measures in fission and budding yeast. (PDF) [file pcbi.1002732.s012.pdf]

Text S1: Predicting essentiality by network measures in fission and budding yeast

Following the work of (Yu *et al*, 2007) on the budding yeast network we analyzed in detail the relationship between degree and betweenness centrality for both networks. Hubs are defined as nodes whose degree is in the top 20% of the distribution. Bottlenecks are defined as nodes whose betweenness centrality is in the top 20% of the distribution. All network measures were calculated with NetworkX (Hagberg *et al*, 2008).

**Fraction of essential genes in:**	**Budding Yeast (%)**	**Fission Yeast %**
Hubs	39	56
Non-hubs	15	30
Bottlenecks	31	47
Non-bottlenecks	17	32
Hubs Non-bottlenecks	44	57
Non-hubs bottlenecks	21	41
Hub Bottlenecks	37	56

Lethality data of budding yeast from (Giaever *et al*, 2002). Lethality data of fission yeast from (Kim *et al*, 2010)

As the relationship gene essentiality and network scores has been explored extensively explored in budding yeast(Yu *et al*, 2007; Coulomb *et al*, 2005; Hahn & Kern, 2005), we repeated some well-established analysis as a sanity check to see if the data for fission yeast gave consistent results. The original analyses in budding yeast were performed on the filtered yeast interactome (FYI), a database containing only very high confidence interactions. Constructing a similar database in fission yeast is currently impossible (such a strict cutoff would result in a very sparse network, see Figure 1), so we repeated the analysis in budding yeast on data from STRING using a cutoff of 0.7. The results of our analysis are consistent with those obtained in the original paper by (Yu *et al*, 2007). We observe that hubs have the highest % of lethal genes in both networks, although hub bottlenecks and hubs non-bottlenecks are very similar.

Next, based on the work of (Coulomb *et al*, 2005; Hahn & Kern, 2005) we examined the ability of different network measures to predict lethality in fission yeast. Further, we checked if genes with a large amount of publication (high number of PubMed abstracts mentioning the gene) were more or less likely to be essential in both organisms.

		**Median:** (Essential, Viable):	**U-Score:**	**p-value:** (Essential, Viable):
**Budding Yeast:**	Degree:	(45, 15)	1197044.5	$< 10^{-117}$
	Betweenness Centrality:	(0.000298, 0.0001)	1568356	$< 10^{-48}$
	PageRank:	(0.000226, 0.000120)	1311455.5	$< 10^{-93}$
	PubMed Count:	(8, 4)	1654226	$< 10^{-37}$
**Fission Yeast:**	Degree	(12, 5)	590746.5	$< 10^{-46}$
	Betweenness Centrality:	(0.000223, 0.000033)	689978	$< 10^{-21}$
	PageRank:	(0.000385, 0.000256)	614423.5	$< 10^{-38}$
	PubMed Count:	(1,1)	865274.5	0.311

For all measures, we show the median score for (essential, viable) genes, the value of the U statistic, and the significance (All p-values from Mann Whitney test, essential vs non-essential genes).

Next, we established the ability of different network measures to predict gene essentiality using logistic regression:

$$f(z) = \frac{1}{1+e^{-z}}$$

Although essential genes differ significantly from non-essential in several network properties, and among the most highly rated genes we observe a higher % of essential genes (see both tables above) we found that network measures were not strong predictors of essentiality (Coulomb *et al*, 2005).

The scaled regression coefficients are:

For **fission yeast**:

$$Z = 0.56 + (1.247 * 10^{-3} * \text{BC}) – (1.294 * 10^{-2} * \text{D}) + (9.89 * 10^{-5} * \text{PR})$$

For **budding yeast**:

$$Z = 1.02 + (1.81 * 10^{-4} * BC) – (3.872 * 10^{-3} * D) + (8.99 * 10^{-5} * PR)$$

Where BC is the scaled betweenness centrality score, D is the scaled degree, and PR is the scaled PageRank. Regressions were calculated using scikits.learn (Pedregosa *et al*, 2011)

References:

Coulomb S, Bauer M, Bernard D & Marsolier-Kergoat M-C (2005) Gene essentiality and the topology of protein interaction networks. *Proceedings. Biological sciences / The Royal Society* **272:** 1721-5

Giaever G, Chu AM, Ni L, Connelly C, Riles L, Véronneau S, Dow S, Lucau-Danila A, Anderson K, André B, Arkin AP, Astromoff A, El-Bakkoury M, Bangham R, Benito R, Brachat S, Campanaro S, Curtiss M, Davis K, Deutschbauer A, *et al* (2002) Functional profiling of the Saccharomyces cerevisiae genome. *Nature* **418:** 387-91

Gkantsidis C, Mihail M & Zegura E (2003) The Markov Chain Simulation Method for Generating Connected Power Law Random Graphs. *Proceedings of the Fifth Workshop on Algorithm Engineering and Experiments***:** 16-25

Hagberg A, Daniel S & Swart P (2008) Exploring Network Structure, Dynamics, and Function using NetworkX. In *Proceedings of the 7th Python in Science conference (SciPy 2008),* Varoquaux G Vaught T, & Millman J (eds) pp 11-15. Pasadena, CA, USA

Hahn MW & Kern AD (2005) Comparative genomics of centrality and essentiality in three eukaryotic protein-interaction networks. *Molecular biology and evolution* **22:** 803-6

Kim D-U, Hayles J, Kim D, Wood V, Park H-O, Won M, Yoo H-S, Duhig T, Nam M, Palmer G, Han S, Jeffery L, Baek S-T, Lee H, Shim YS, Lee M, Kim L, Heo K-S, Noh EJ, Lee A-R, *et al* (2010) Analysis of a genome-wide set of gene deletions in the fission yeast Schizosaccharomyces pombe. *Nature biotechnology* **28:** 617-23

Pedregosa F, Varoquaux G, Gramfort A, Michel V, Thirion B, Grisel O, Blondel M, Prettenhofer P, Weiss R, Dubourg V, Vanderplas J, Passos A, Cournapeau D, Brucher M, Perrot M & Duchesnay E (2011) Scikit-learn: Machine Learning in Python. *Journal of Machine Learning Research* **12:** 2825--2830

Yu H, Kim PM, Sprecher E, Trifonov V & Gerstein M (2007) The importance of bottlenecks in protein networks: correlation with gene essentiality and expression dynamics. *PLoS computational biology* **3:** e59
