# Supplementary material for: Linkers of Cell Polarity and Cell Cycle Regulation in the Fission Yeast Protein Interaction Network
Source: PLoS Comput Biol. 2012 Oct 18;8(10):e1002732. doi: 10.1371/journal.pcbi.1002732 (PMC3475659; doi:10.1371/journal.pcbi.1002732)
Supplement: Text S2 — Analysis of the neighbors of high linkerity proteins. (PDF) [file pcbi.1002732.s013.pdf]

Text S2: Analysis of the neighbors of high linkerity proteins

We tested the relationship between the linkerity of a node in the network and the functional annotation of its neighbors. To do this, for every node with a given functional annotation, we calculated the ratio of its interactions with neighbors sharing a functional annotation versus neighbors with different functional annotations. For neighbors with multiple functional annotations, we counted each functional annotation individually.

For example, looking at a simplified network:

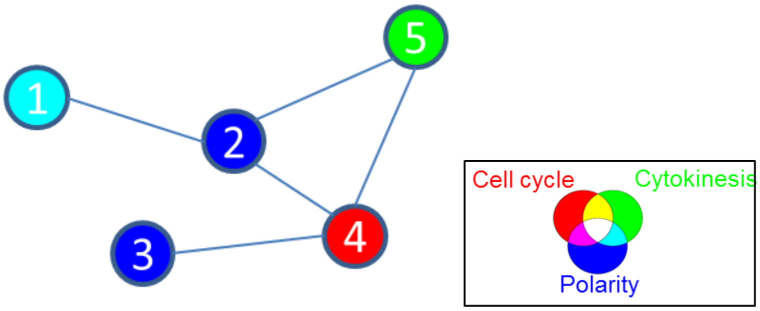

Given node 2 with a polarity functional annotation (in blue), and four neighbours (1, 3, 4, 5) we obtain:

Self annotations:

2-1 (Polarity to Polarity)

Non-self annotations:

2-1 (Polarity to Cytokinesis)

2-5 (Polarity to Cytokinesis)

2-4 (Polarity to Regulation of Cell Cycle)

Giving a ratio of 1/3.

We then calculated the Spearman correlation between the other/self interaction ratio and the linkerity measure for the proteins in every functional category.

	**Budding Yeast**		**Fission Yeast**	
	**Spearman Corr:**	**p-value**	**Spearman Corr:**	**p-value**
**Polarity:**	**0.49**	**$<10^{-9}$**	**0.60**	**$<10^{-9}$**
**Cell Cycle:**	**0.42**	**$<10^{-8}$**	**0.30**	**$<10^{-7}$**
**Cytokinesis:**	**0.42**	**$<10^{-4}$**	**0.36**	**$<10^{-4}$**
