# Supplementary material for: Linkers of Cell Polarity and Cell Cycle Regulation in the Fission Yeast Protein Interaction Network
Source: PLoS Comput Biol. 2012 Oct 18;8(10):e1002732. doi: 10.1371/journal.pcbi.1002732 (PMC3475659; doi:10.1371/journal.pcbi.1002732)
Supplement: Text S3 — Correlation between linkerity and other network measures. (PDF) [file pcbi.1002732.s014.pdf]

Text S3: Correlations between linkerity and other network measures

We calculated the Spearman correlation (SC) between various network indices in each of the subnetworks analyzed in the paper. Betweenness and degree are highly correlated in all subnetworks. Linkerity is not correlated with any degree or BC, although it shows a significant correlation to the BC of the corresponding node in the core network.

Budding Yeast:

Cell Cycle:

	Degree:		Linkerity:	
	p-value:	SC:	p-value:	SC:
Betweenness Centrality:	$< 10^{-42}$	0.8	.54	-0.04
Degree:			.95	.004

Core Betweenness Centrality and Linkerity Correlation: 0.36, p-value: $1.4 * 10^{-7}$

Cytokinesis:

	Degree:		Linkerity:	
	p-value:	SC:	p-value:	SC:
Betweenness Centrality:	$< 10^{-15}$	0.68	.38	-0.9
Degree:			.90	-0.01

Core Betweenness Centrality and Linkerity Correlation: 0.48, p-value: $3 * 10^{-7}$

Polarity:

	Degree:		Linkerity:	
	p-value:	SC:	p-value:	SC:
Betweenness Centrality:	$< 10^{-30}$	0.8	.37	-0.08
Degree:			.88	-0.012

Core Betweenness Centrality and Linkerity Correlation: 0.29, p-value: 0.0005

Fission Yeast:

Cell Cycle:

	Degree:		Linkerity:	
	p-value:	SC:	p-value:	SC:
Betweenness Centrality:	$< 10^{-45}$	0.68	.46	-0.04
Degree:			.41	-0.04

Core Betweenness Centrality and Linkerity Correlation: 0.13, p-value: 0.01

Cytokinesis:

	Degree:		Linkerity:	
	p-value:	SC:	p-value:	SC:
Betweenness Centrality:	$< 10^{-14}$	0.65	.60	-0.05
Degree:			.20	0.192

Core Betweenness Centrality and Linkerity Correlation: 0.47, p-value: $1.5 * 10^{-7}$

Polarity:

	Degree:		Linkerity:	
	p-value:	SC:	p-value:	SC:
Betweenness Centrality:	$< 10^{-29}$	0.9	.23	-0.13
Degree:			.29	-0.11

Core Betweenness Centrality and Linkerity Correlation: 0.35, p-value: 0.0085
