# Supplementary material for: Linkers of Cell Polarity and Cell Cycle Regulation in the Fission Yeast Protein Interaction Network
Source: PLoS Comput Biol. 2012 Oct 18;8(10):e1002732. doi: 10.1371/journal.pcbi.1002732 (PMC3475659; doi:10.1371/journal.pcbi.1002732)
Supplement: Text S4 — Linkerity at various network confidences. (PDF) [file pcbi.1002732.s015.pdf]

Text S4: Linkerity at various network confidences

In Supplementary Tables 3 and 4, we show the linkerity values for all proteins calculated using the STRING database with a confidence cutoff of 0.7. Here, we examine in details the relationship between the stringency of the cutoff applied to the interaction data and linkerity.

To do this, we created networks using STRING cutoffs of 0.4, 0.7, 0.9, and BioGRID physical interaction), and calculated the Spearman correlation between the linkerity values obtained in the different confidence networks.

Fission Yeast Polarity Linkerity Correlation:

Cutoff1: **BioGRID** *physical*	Cutoff2: **STRING** *0.4*	Correlation: 0.0357	p-value < 0.80
Cutoff1: **BioGRID** *physical*	Cutoff2: **STRING** *0.7*	Correlation: 0.0114	p-value < 0.94
Cutoff1: **BioGRID** *physical*	Cutoff2: **STRING** *0.9*	Correlation: 0.38	p-value < 0.053
Cutoff1: **STRING** *0.4*	Cutoff2: **STRING** *0.7*	Correlation: 0.579	p-value < 5.224e-09
Cutoff1: **STRING** *0.4*	Cutoff2: **STRING** *0.9*	Correlation: 0.172	p-value < 0.354
Cutoff1: **STRING** *0.7*	Cutoff2: **STRING** *0.9*	Correlation: 0.129	p-value < 0.489

Budding Yeast Polarity Linkerity Correlation:

Cutoff1: **BioGRID** *physical*	Cutoff2: **STRING** *0.4*	Correlation: 0.74	p-value < 1.25e-24
Cutoff1: **BioGRID** *physical*	Cutoff2: **STRING** *0.7*	Correlation: 0.67	p-value < 6.373e-18
Cutoff1: **BioGRID** *physical*	Cutoff2: **STRING** *0.9*	Correlation: 0.589	p-value < 9.63e-12
Cutoff1: **STRING** *0.4*	Cutoff2: **STRING** *0.7*	Correlation: 0.801	p-value < 1.42e-32
Cutoff1: **STRING** *0.4*	Cutoff2: **STRING** *0.9*	Correlation: 0.67	p-value < 8.987e-17
Cutoff1: **STRING** *0.7*	Cutoff2: **STRING** *0.9*	Correlation: 0.885	p-value < 5.258e-40

In the case of the better characterized budding yeast data we see high correlation between all values, while in the less well characterized fission yeast network the correlation is significant only between STRING at cutoff 0.4 and 0.7. Interestingly even STRING 0.9 and BioGRID physical do not show real strong correlation in this organism.
